# Molecular Structure, Spectral Analysis, Molecular Docking and Physicochemical Studies of 3-Bromo-2-hydroxypyridine Monomer and Dimer as Bromodomain Inhibitors

**DOI:** 10.3390/molecules28062669

**Published:** 2023-03-15

**Authors:** Nizar Lefi, Aleksandr S. Kazachenko, Murugesan Raja, Noureddine Issaoui, Anna S. Kazachenko

**Affiliations:** 1 Department of Physics, College of Sciences and Arts in Uglat Asugour, Qassim University, Buraydah 52571, Saudi Arabia; 2 Laboratory of Quantum and Statistical Physics (LR18ES18), Faculty of Sciences, University of Monastir, Monastir 5079, Tunisia; 3 Institute of Chemistry and Chemical Technology, Krasnoyarsk Scientific Center, Siberian Branch, Russian Academy of Sciences, Akademgorodok 50, Bld. 24, 660036 Krasnoyarsk, Russia; 4 Department of Organic and Analytical Chemistry, Siberian Federal University, pr. Svobodny 79, 660041 Krasnoyarsk, Russia; 5 Department of Biological Chemistry with Courses in Medical, Pharmaceutical and Toxicological Chemistry, Krasnoyarsk State Medical University, St. Partizan Zheleznyak, Bld. 1, 660022 Krasnoyarsk, Russia; 6 Department of Physics, Govt. Thirumagal Mills College, Gudiyatham, Vellore 632602, India

**Keywords:** density functional theory, 3-bromo-2-hydroxypyridine, HOMO–LUMO, MEP, RDG, molecular docking

## Abstract

In this paper, both methods (DFT and HF) were used in a theoretical investigation of 3-bromo-2-Hydroxypyridine (3-Br-2HyP) molecules where the molecular structures of the title compound have been optimized. Molecular electrostatic potential (MEP) was computed using the B3LYP/6-311++G(d,p) level of theory. The time-dependent density functional theory (TD-DFT) approach was used to simulate the HOMO (highest occupied molecular orbital) and LUMO (lowest unoccupied molecular orbital) on the one hand to achieve the frontier orbital gap and on the other hand to calculate the UV–visible spectrum of the compound in gas phase and for different solvents. In addition, electronic localization function and Fukui functions were carried out. Intermolecular interactions were discussed by the topological AIM (atoms in molecules) approach. The thermodynamic functions have been reported with the help of spectroscopic data using statistical methods revealing the correlations between these functions and temperature. To describe the non-covalent interactions, the reduced density gradient (RDG) analysis is performed. To study the biological activity of the compound of the molecule, molecular docking studies were executed on the active sites of BRD2 inhibitors and to explore the hydrogen bond interaction, minimum binding energies with targeted receptors such as PDB ID: 5IBN, 3U5K, 6CD5 were calculated.

## 1. Introduction

Heterocyclic compounds with nitrogen, which are derived by replacing a carbon atom in the ring with a nitrogen atom, have attracted considerable research interest over the past century because of their extensive physiochemical properties [1,2]. Heterocyclic organic compounds such as pyridine are structurally similar to benzene but are substituted with nitrogen instead of C–H. Pyridine and its derivatives play an important role in large number of applications ranging from medicinal drugs to agriculture products [3,4,5,6] and enzyme inhibitors [7,8]. A survey of the literature reveals that substituted pyridine derivatives exhibit various studies of the halogen-substituted pyridines. Some of these are listed below. Vibrational assignments for 2-iodopyridine have been proposed based on the FT-Raman and IR spectra [9]. Vibrational spectroscopy based on FTIR and FT-Raman spectra measured in the condensed state has been reported for 2,6-dichloropyridine [10]. The solid phase FTIR and FT-Raman spectra of di- and tri-halopyridines were represented [11,12]. 2-Chloro-5-bromopyridine was investigated by spectroscopic studies, normal coordinate analysis and electronic structure analysis [13]. The ultraviolet absorption spectrum in the vapor and solution phase has been measured for 2-fluoro-5-bromopyridine [14]. In this study, HF and DFT studies of the 3-bromo-2-hydroxypyridine was performed. Thus, to fulfill the inadequacy observed in the literature concerning some pyridine derivatives inspired us to choose in a first step the 3-bromo-2-hydroxypyridine in this work that can subsequently be extended for huge- and medium-sized molecules of pyridine derivatives. So, to understand the in-depth insight of the title molecule, a theoretical study of the 3-bromo-2-hydroxypyridine molecule has been undertaken. The IUPAC name of 3-bromo-2-hydroxypyridine is 3-bromo-1H-pyridin-2-one, and it is also named as 2-Hydroxy-3-bromopyridine. This molecule exists as a light-yellow powder. M. Pervaiz et al. [15] and A. Alqahtani et al. [16] reported the significance of the bromodomain inhibitors (BRD) inhibitors, which play a substantial role in the identification of novel drug targets and are specially being evaluated clinically across a range of cancers, inflammation, obesity and cardiovascular diseases.

For the polyatomic molecules and to understand the molecular properties and bonding in compounds, the optimized molecular parameters are calculated using the computational molecular modeling tools such as ab initio and density functional theory (DFT). For large- and medium-size molecules an excellent compromise between accuracy and computational efficiency of vibrational spectra has been reported for the use of Beck’s three parameter hybrids functional combined with the Lee–Yang–Parr correlation functional (B3LYP) [17,18,19,20]. Recently, DFT is becoming more useful to experimentalists than the conventional ab initio Hartree–Fock calculations for the prediction of geometrical parameters and some other properties such as vibrational frequencies, dipole moment, etc. [21,22,23,24,25].

In this paper, diverse properties of 3-Br-2-HyP are investigated using both methods, namely B3LYP and HF. We concerned the distribution of electrons and the reactive sites on the surface of the title molecule by analyzing the molecular electrostatic potential (MEP), electron localization function (ELF) and localized orbital locater (LOL). To elucidate information regarding charge transfer within the molecule, the frontier molecular orbital analysis was used. AIM theory [26,27,28] was used to calculate the topological parameters and a conscientious attention has been focused to investigate the effects of intermolecular O–H···N hydrogen bonding on the distances and nature of these bonds. The thermodynamic proprieties (such as thermal energy, specific heat capacity at constant volume, entropy and dipole moment) have been calculated and reported. The possible non-covalent interactions were visualized in reduced density gradient (RDG) analysis [29]. The temperature dependence of thermodynamic properties has been analyzed. The local reactivity descriptors such as Fukui functions were plotted. The molecular docking study was employed to envisage the potential biological activity of the title compound.

## 2. Computational Details

In order to model the molecular structure and to calculate the interaction energy, the Gaussian 09 software package [30] was used based on DFT and ab initio level [31,32]. To confirm the optimized geometry of the title molecule, all stationary points are identified as minima. A triple split valence basis set along with a diffuse and polarization functions 6-311++G(d,p) basis set was used in all the calculations on the investigated systems. In order to arrive at the minimum energy conformer of the title molecule, we have scanned the total energy by varying the N–C–O–H dihedral angle and optimizing the remaining molecular parameters for each fixed dihedral angle using the DFT/B3LYP/6-311++(d,p) level of theory. The optimized molecular geometry, HOMO, LUMO energy distributions and HOMO–LUMO energy gap [33,34] have been constructed using the GaussView 5.0.8 program [35]. The molecular electrostatic potential has been performed to investigate the reactive sites of the title molecule. Furthermore, the AIM2000 program [36] was used to realize the AIM computations for describing the possibility of intermolecular hydrogen bonds.

In this study, the TD-DFT is proven to be a powerful and effective computational tool. The B3LYP method of time-dependent TD-DFT, based on the optimized structure in the gas phase and solvents (DMSO, water, methanol and chloroform), was used to calculate the energies, absorption wavelengths and oscillator strengths. The same method, TD-DFT with the 6-311++G(d,p) basis set, was used to obtain the theoretical UV–Vis spectrum. Moreover, to identify the non-covalent interactions, the reduced density gradient (RDG) of the studied compound was performed using the Multiwfn package [37], and RDG plots were generated through the visual molecular dynamics program (VMD) [38]. The changes in the thermodynamic functions (heat capacity, entropy and enthalpy) were computed for the different temperatures of title molecule. As the ELF and LOL reveal regions of molecular space where the probability of finding an electron pair is higher, they are used to perform covalent bonding analysis [39,40]. To study the chemical selectivity or reactivity at a particular site of a chemical system the Fukui functions, which describe the electron density-based local reactivity, are proposed [41]. The ligand (title compound) and targeted protein docking simulations are performed using AutoDock 4.2.6 software.

## 3. Results and Discussion

### 3.1. Geometric Structure

**Figure 1:**
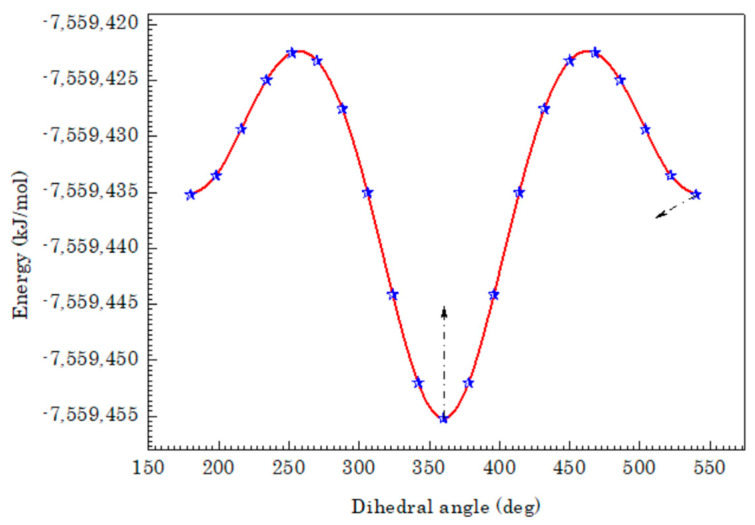
The PE curve of 3-Br-2-HyP along the N5–C2–O6–H10 dihedral angle.

The first task for the computational work was to obtain the most energetical conformation of the molecule and the optimized geometry will be confirmed with configuration of minimum energy of the compound. The procedure that was followed to visualize and describe the relationship between potential energy and molecular geometry is the potential energy surface. So, the most stable geometry of the 3-Br-2-HyP molecule was found by performing conformational analysis through the potential surface scan. The potential energy surface curve versus with dihedral angle N5–C2–O6–H10 was carried out for 3-Br-2-HyP molecule using the B3LYP/6-311++G(d,p) level of theory. Figure 1 illustrates the potential energy curve of the title compound as a function of the dihedral angle N5–C2–O6–H10. It can be seen from Figure 1 that at 360°, the local minimum was observed and the corresponding potential energy is equal to −2879.2422 Hartrees. Thus, the optimized geometry corresponding to the dihedral angle, N5–C2–O6–H10 of 360° in the potential energy curve belongs to the most stable geometry of the studied compound.

**Figure 2:**
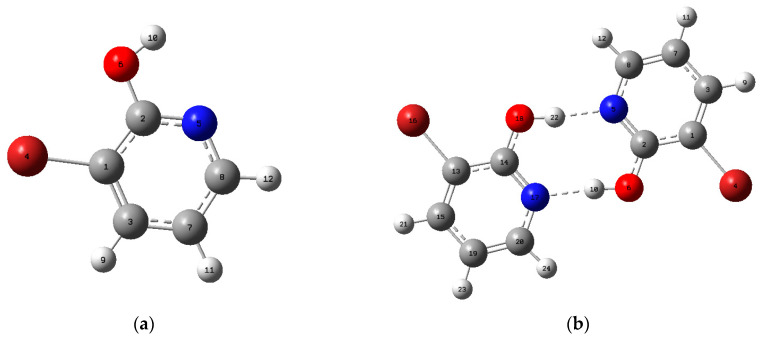
Theoretical optimized geometry of the 3-Br-2-HyP molecule of the monomer (**a**) and dimer (**b**) with labeling atoms calculated by DFT method.

The optimized molecular structures of the 3-Br-2-HyP monomer and dimer with the numbering scheme of the atoms are presented in Figure 2, whereas the optimized geometrical parameters of the 3-Br-2-HyP monomer and dimer were calculated using the HF and B3LYP method with the 6-311++G(d,p) basis set. The corresponding results of the geometrical parameters are gathered in Table 1 and compared with those of available similar systems [13,42]. Similar to what was as observed in Figure 2 for dimer structure and by analyzing the theoretical data obtained, it was found that a strong intermolecular O–H···N hydrogen bond of 1.7163 Å has appeared between the N5(17) and H10(22) atoms. This intermolecular H-bond gives rise to a dimer structure of the title compound. Referring to Table 1, the computed bond distances are equal to 1.0073 Å (O–H), 1.7163 Å (O–H···N) and 2.7236 Å (O···N). The O–H···N bond angle is equal to 174.159°. When comparing the optimized and the experimental geometrical values, we conclude that calculated bond lengths and angles are slightly longer and shorter than the experimental values. This discrepancy may be explained by the fact that the experimental results belong to molecules in the solid state and the theoretical calculations belong to isolated molecules in gaseous phase. Comparing the computed geometrical parameters in terms of the bond angles and bond lengths of B3LYP with those of HF, it is evident that the B3LYP calculated values correlate better with the experimental data than with those obtained using HF method. In the present molecule, for both the HF and DFT methods the correlation equation between the optimized geometrical parameters and available experimental values of a similar system are fitted by correlation coefficients and the corresponding fitting factors (R2). In Figure S1, we have reported the values and corresponding correlation graphics. These fitting factors (R2) are 0.99773, 0.99857, 0.94212 and 0.94872, respectively.

In spite of the differences, computed geometrical parameters lead to a good approximation and can be applied for calculating vibrational frequencies and thermodynamic properties. The values of thermodynamic functions are calculated using the B3LYP method with the 6-311++G(d,p) basis set at 298.15 K and listed in Table S1. As follows from the Table S1, for the title compound the global minimum energy calculated by the DFT method is −7,606 473.06 kJ/mol and −15,213 012.632 kJ/mol in the monomer and dimer structures, respectively. As shown in Figure 2, the interaction in the dimer structure of 3-Br-2-HyP presents two equivalent, stable, hydrogen-bonded O–H···N contacts (O18–H22···N5 and N17···H10–O6) that result in increased stabilization. The interaction energy on dimer formation through the doubly hydrogen-bonded interface was calculated using the super molecule method [43,44]. The hydrogen-bonded interaction energy of the formation of a 3-Br-2-HyP dimer is calculated as the difference between the computed total energy of the dimer structure and the energies of the two isolated monomers. The value of the interaction energy of the dimer structure for the title molecule is calculated and found to be 15.896 kcal/mol. The stability of the 3-Br-2-HyP molecule can be explained by the high values of the dipole moment (2.1152 Debye), which favor the formation of the inter-molecular H-bond dimer.

### 3.2. Molecular Electrostatic Potential

**Figure 3:**
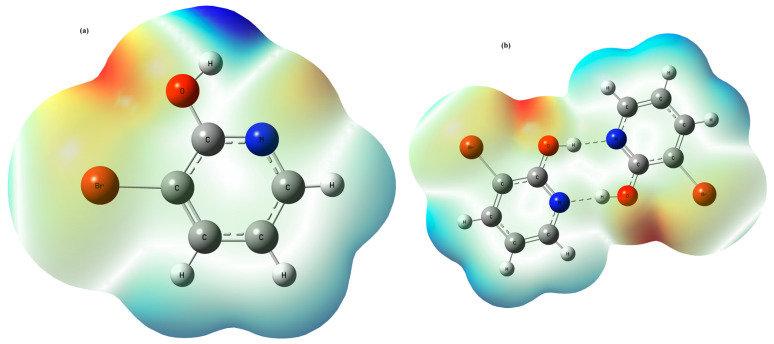
3D molecular electrostatic potential contour map of 3-Br-2-HyP monomer (**a**) and dimer (**b**) calculated at B3LYP/6-311++G(d,p) level.

In the subject to comprehend the relative polarity [45,46], the behavior and reactivity of the molecules of the MEP surfaces are used as a visual method. Its mapping potentials illustrate the charge distributions of molecules three dimensionally. MEP is powerful method that gives qualitative interpretation of the electrophilic and nucleophilic reactions for the study of the biological recognition process and intermolecular interaction such as H-bonding interactions [47,48]. This later study of the interacting behavior of various constituents of the molecule has great importance in hydrogen-bonded systems. This also plays a vital role for the researchers to provide information and to understand the physiochemical property of the molecules such as size, shape, charge density, delocalization and site of chemical reactivity. The DFT/B3LYP/6-311++G(d,p) method was employed to construct the total electron densities of the 3-Br-2-HyP monomer and dimer structures; the generated maps are shown in Figure 3. The MEP surfaces of the monomer and the dimer of the studied compound are mapped throughout a color code ranging from −3.782 × 10^−2^ to 3.782 × 10^−2^. Potential increases in the order red < orange < yellow < green < blue. In all compounds, the blue color of these maps illustrates the strongest attraction, and the red color indicates the strongest repulsion. The lone pair of electronegative atoms is usually associated with the regions of negative electrostatic potential. From Figure 3, the MEP map of the title molecule shows two types of regions: the first one with negative potential is over the electronegative atoms (oxygen, nitrogen and bromine), whereas the second with the positive potential is over the hydrogen atoms. From the MEP surface calculated for the monomer, the strong blue colors are observed on hydrogen atoms (H9, H11, H10 and H12). For the dimer structure of the title compound, due to the greater molecular electrostatic potential values, strong red colors appeared on the oxygen and nitrogen atoms. From the above analysis, the greater molecular electrostatic potential values for the pairs of N5 and O6 and N17 and O18 atoms can explain the electrostatic repulsion between these two pairs. For both the molecular electrostatic potential maps of the title molecule monomer and dimer, it is evident from Figure 3 that the observed green areas cover parts of the molecule where the electrostatic potentials are close to zero, which characterizes C–C and C–H bonds.

### 3.3. Topological Parameters

**Figure 4:**
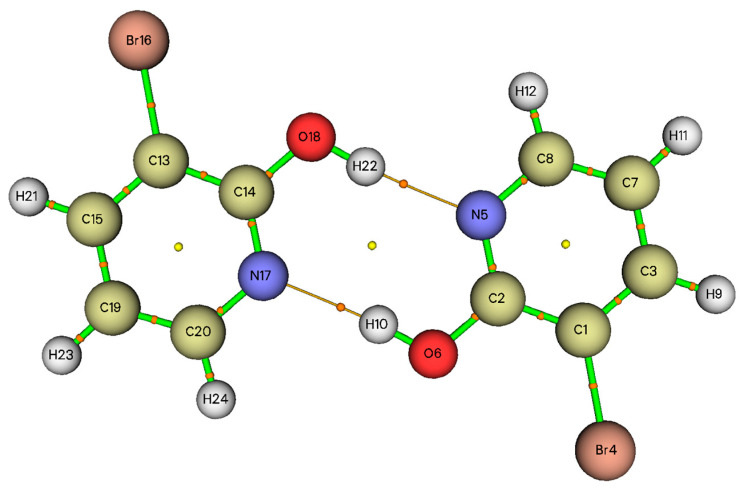
Molecular graphic for 3-Br-2-HyP dimer.

Much theoretical research shows that the AIM theory proposed by Bader is the most useful tool to investigate the bonding nature of weak interactions in various molecular systems, principally hydrogen bonding [26,27,28]. According to this theory, the existence of the bond critical points (BCPs) is the key to characterize any chemical bond including hydrogen bonding. Many properties can be computed at their position in space after the localization of the bond critical points. According to these properties, the charge density $\rho_{BCP}$ and the Laplacian of the charge density $\nabla^{2}\rho_{BCP}$ at BCP are very important parameters. In the context of AIM analysis, Popelier and Koch developed eight topological parameters for the existence of H-bonding interactions (HBIs) [49], whereas only the following criteria for them were widely useful to characterize hydrogen bonds: (i) the existence of HBIs is confirmed by the presence of BCP between the proton (H) and acceptor (X), (ii) the value of $\rho_{BCP}$ and the corresponding $\nabla^{2}\rho_{BCP}$ at BCP should lie in the ranges 0.002–0.040 a.u and 0.024–0.139 a.u, respectively. The AIM program [36] is used to observe the molecular graph of the dimer structure of the title molecule and the generated graph is provided in Figure 4. The critical point properties for various non-covalent, covalent and H-bonds are summarized in Table 2.

As can be seen in Table 2, The BCPs located in N5···H22 and H10···N17 hydrogen bonds are characterized by low charge density (0.052 a.u.) and a positive Laplacian of electron density (0.0998 a.u.), which are closer to the values proposed by the Popelier and Koch criteria. Positive values of Laplacian $\nabla^{2}\rho_{BCP}$ denote the depletion of electronic charge along the bond path, which is characteristic of closed-shell interactions and can be used to distinguish between shared-shell and closed-shell bonding interactions. The interaction energy at BCP is obtained using the relationship proposed by Espinosa et al. [50], defined as follows:

(1) $$E_{HB}=0.5V_{BCP},$$

where $V_{BCP}=1/4\nabla^{2}\rho_{BCP}–2G$ is the potential energy electrostatic density at the bond critical points and G is the local electron energy density. In this case of the 3-Br-2-HyP dimer, the $E_{HB}$ has been calculated by the B3LYP method to be 66.291kJ/mol, which confirms the existence of strong hydrogen bonding [51,52,53]. One can acquire to find the knowledge about characteristics of the non-covalent interaction (H-bonding) through the energetic properties of BCP associated with the interaction. Among these, the ratio $G/\rho \left(r_{C}\right)$ can be used to define the character of the interaction which, for closed shells such as H-bonding, vdr interaction and ionic bonds where this ratio may be larger than unit [26]. Furthermore, the results in Table 2 show that the bond critical points found in the inter-H-bonding are associated with two negative (λ_1_ = −0.0914 and λ_2_ = −0.0870) and one positive eigenvalues (λ_3_ = 0.2783). At the BCPs located in N5···H22 and H10···N17 H-bonds, the value of |λ_1_|/λ_3_ is equal to 0.3284, which in general arises less than unit in HBIs.

### 3.4. Frontier Molecular Orbitals

The way that the molecule interacts with other species can be predict by the highest occupied molecular orbital (HOMO) and lowest unoccupied molecular orbital (LUMO). The frontier orbital gap is the energy difference between the HOMO and LUMO. This is an important index for characterizing the kinetic stability and the chemical reactivity of the molecule. A small frontier orbital gap means that the molecule is polarizable, reactive and is a soft molecule, whereas a large frontier orbital gap means a hard molecule [54,55,56]. The frontier molecular orbitals are significant for optical and electric properties [57]. The LUMO represents the ability to obtain an electron and HOMO, as an electron donor, shows the ability to give an electron [58]. The frontier orbital gap was used not only to study the stability of structures but also to investigate the molecular electrical transport properties in terms of the measurement of electron conductivity [59,60]. In Table 3, we report the calculated parameters for the monomer structure using the TD–DFT method in the gas phase and in many solvents.

The computed energy values of HOMO are −6.879, −6.880, −6.880 and −6.880 eV in methanol, water, DMSO and gas, respectively. The calculated values of LUMO are −1.475, −1.475, −1.475 and −1.475 eV in methanol, water, DMSO and gas, respectively. The values of the gap are found energy separation between the HOMO and LUMO are 5.403, 5.406, 5.405 and 5.406 eV in methanol, water, DMSO and gas, respectively.

**Figure 5:**
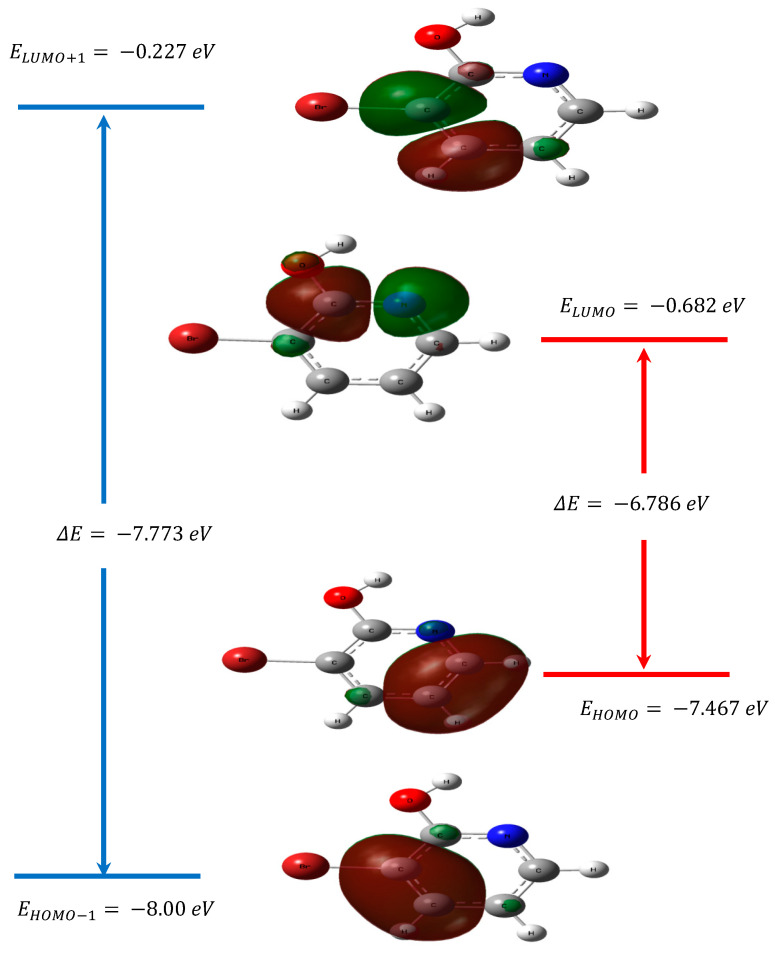
The first and second frontier molecular orbitals with the energy gap of 3-Br-2-HyP molecule.

To well understand the H-bonding of a studied molecule, the surfaces for the Frontier orbitals were depicted and we focused on the four most important molecular orbitals (MO) for the title compound, especially on the second highest and highest occupied MOs (HOMO − 1 and HOMO) and the lowest and the second lowest unoccupied ones (LUMO and LUMO + 1). The 3D plots of these Frontier orbitals for the studied molecule are illustrated in Figure 5. As can be seen in Figure 5, the energy of the HOMO level is −7.467 eV and the HOMO displays with a delocalized π character that is located around the all C atoms of pyridine. The LUMO energy level is −0.682 eV, and the LUMO orbitals are $\pi^{*}$ in type. The HOMO→LUMO transition implies an electron density transfer to the whole pyridine ring and oxygen atom. As a result, the energy gap between HOMO and LUMO was calculated as −6.785 eV. Hence the probability of $\pi \to \pi^{*}$ proton transition is highly possible between HOMO and LUMO orbitals of 3-Br-2-HyP, which can be explained by the very small observed energy gap.

### 3.5. UV–Visible Spectral Analysis

All compounds show strong $\pi \to \pi^{*}$ and $\sigma \to \sigma^{*}$ transitions in the UV–Vis spectrum high extinction coefficients [61,62]. The TD–DFT method is a most common approach that is computationally reasonable and allows study of medium size molecules [63,64,65]. The simulated excitation energies (E), oscillator strength (*f*), wavelength of absorption (λ) and percentage of molecular orbital contribution in the transitions have been theoretically obtained in the framework of TD-DFT calculation with the B3LYP/6-311++G(d,p) method; these are given in Table 4, and to the best of our knowledge, no experimental UV absorption spectra of 3-Br-2-HyP were recorded. The major contributions were assigned with the help of the SWizard program [66]. For the 3-Br-2-HyP compound, from the results of Table 4, we can note the appearance of intense electronic transition at 260.3 nm with an oscillator strength of 0.1431 in gas phase, 260.8 nm (*f* = 0.1506) in DMSO, 260.3 nm (*f* = 0.1431) in water, 260.4 nm (*f* = 0.1428) in methanol and 261.9 nm (*f* = 0.1541) in chloroform. This electronic absorption corresponds to the one electron excitation from the HOMO to the LUMO+1, whereas in the gas phase the maximum absorption wavelength corresponds to the electronic transition from the HOMO to the LUMO+1 with a 62% contribution and the transition from the HOMO to the LUMO with a 91% contribution. Thus, the negative orbital energy of the HOMO and the energy of the LUMO provide the first ionization energy and electron affinity of a molecular system, respectively.

### 3.6. Reduced Density Gradient (RDG)

RDG is one of the most relevant techniques used to study the non-covalent interactions in real space based on the electron density and its derivatives. This technique represents a fundamental dimensionless quantity in DFT used to describe the deviation from electron distribution. In 2010, the RDG approach was published by Johnson et al. [29]. This approach was demonstrated to be capable of distinguishing hydrogen bonds, Van der Waals interactions and steric repulsion in small molecules, molecular complexes and solids. To calculate the RDG quantity, we use the following expression:

(2) $$RDG\left(r\right)=\frac{1}{2{\left(3\pi^{2}\right)}^{1/3}}\frac{\left|\nabla \rho \left(r\right)\right|}{{\rho \left(r\right)}^{4/3}},$$

**Figure 6:**
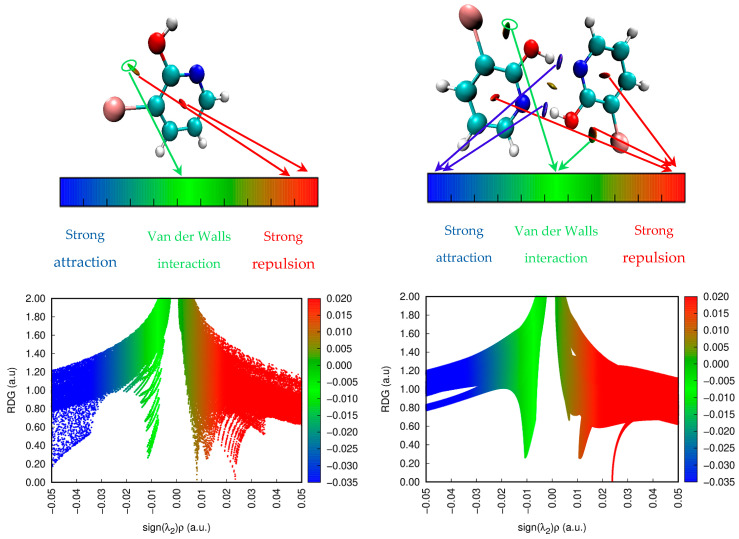
RDG function iso-surface with sign (λ_2_)ρ coloring scheme for crystalline structure of 3-Br-2-HyP molecule.

By analyzing the low-density gradient, the low electron region can be confirmed, which is responsible for weak interactions. Similarly, the high-density gradient value is used to identify the strong interactions. The plots of RDG versus ρ (density values of the low-gradient spike) appear to be an indicator of the interaction strength. The product of electron density ρ and the sign of the second eigenvalues λ_2_ (second largest value of hessian matrix) has been proposed as a tool to distinguish a wide range of interaction types. The sign of λ_2_ is critical parameter used to differentiate between bonded (λ_2_ < 0) and non-bonded (λ_2_ > 0) interactions. RDG was calculated using the Multiwfn program [37] and plotted by the VMD program [38]. Basing on the sign(λ_2_)ρ, the kind of interactions can be classified into three types [67]. The first represents attractive attraction (hydrogen bonding or dipole–dipole) and it is characterize by negative values of sign(λ_2_)ρ. The second suggests non-bonding interaction such as strong repulsion, which is confirmed by large and positive values of sign (λ_2_)ρ. The third type, namely weak interactions (Van der Waals interactions), are associated with values near zero. In Figure 6, we report the predicted RDG plot for the monomer and dimer of the title molecule. As shown in Figure 6, the plot can be divided in two parts where the left one indicates (λ_2_ < 0) and the right one is associate with (λ_2_ > 0). The interaction between the two entities forming the dimmer molecules shows that three types of interactions are taking place. The first one in the blue color between the hydroxyl group and nitrogen atom corresponds to the strong hydrogen bond interactions. The second one in the red color for both the monomer and dimer in the ring system denotes strong repulsion. The third region in green shows the Van der Waals interactions. The existences of these types of interactions in the 3-Br-2-HyP molecule proves the stability of the molecular system.

### 3.7. Thermodynamic Properties

For the working molecule, the computational harmonic frequencies were used to study the variation in thermodynamic parameters viz., heat capacity at constant pressure (C_p_), entropy (S) and enthalpy (H), for a temperature range 100–1000 K. The obtained results are tabulated in Table 5 and plotted in Supplementary Materials Figure S2. The thermodynamic functions rise linearly when the temperature increases from 100 to 1000 K. The behavior of the thermodynamic functions may be explained by the variation of the molecular vibrational intensities with temperature [68,69]. The correlation equations between heat capacity at constant pressure (C_p_), entropy (S) and enthalpy (H) and temperature are fitted with quadratic formulae. The corresponding fitting factors (R2) of each thermodynamic quantity are 0.99865, 0.99999, and 0.99981, respectively.

(3) $$C_{p}=15.21375+0.37722\times T-1.69232\times 10^{-4}\times T^{2},$$

(4) $$S=230.1454+0.43457\times T-1.08958\times 10^{-4}\times T^{2},$$

(5) $$H=-12.63881+0.08387\times T+7.67738\times 10^{-4}\times T^{2},$$

The thermodynamic parameters of the title compound are computed using the DFT method and are reported in Table S1. For further study on the title compound, all the above-mentioned thermodynamic data may be used not only to calculate the other thermodynamic parameters according to the relationships of thermodynamic functions but also to estimate the directions of chemical reactions according to the second law of thermodynamics [69,70].

### 3.8. ELF and LOL Analysis

**Figure 7:**
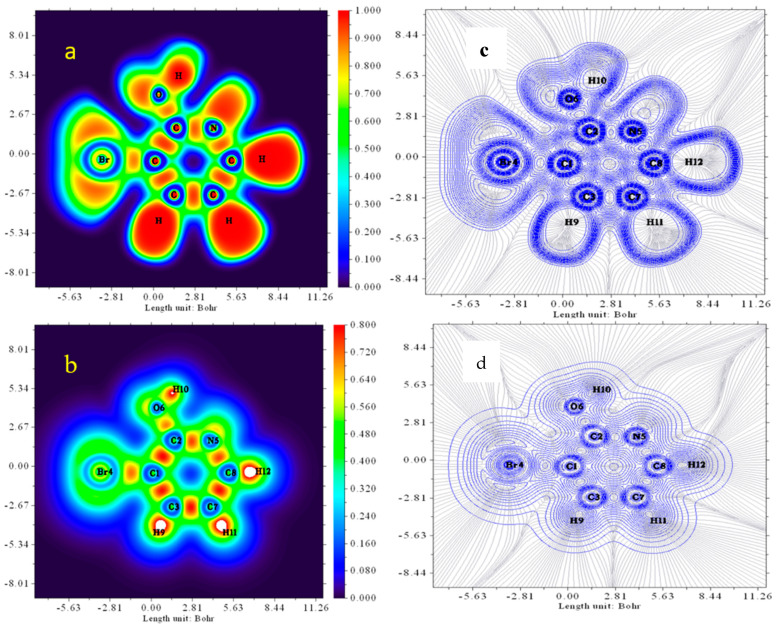
ELF (**a**,**c**) and LOL (**b**,**d**) of title compound.

To gain a better understanding of covalent bonding, we performed topological analyses of the electron localization function (ELF) and localized orbital locator (LOL), which reveal regions with a high probability of finding a pair of electrons [39]. The contour line map is one of the representations to visualize the three-dimensional nature of the compound. Recall that the electron localization function and localized orbital locator generally have a similar qualitative comparison where ELF reveals a relative localization that is generalized for a spin-polarized system and has direct correlation with Femi hole integration and LOL gives the actual kinetic energy term and conveys a more decisive and clearer picture than ELF. The ELF and LOL analysis was achieved on the geometrically optimized structures using the Multiwfn software package [37]. The ELF and LOL maps of the study compound molecule are depicted in the color shade map and contour map that are presented in Figure 7. The ELF values range from 0.0 to 1.0 and relatively specify the region containing bonding and non-bonding localized electrons in the interval 0.5 and 1.0; the delocalized region is expected to be in the smaller values (less than 0.5). By examining the results observed in Figure 7, the high ELF region (in red) around hydrogen atoms and blue color region with carbon atoms specifies the delocalized electron cloud around it. The color map of LOL shows the white color at the central part of the hydrogen atom denotes the electron density is higher than the color scale limit represented in the color scale map (0.80) [40]. The bromine and a few carbon nuclei indicate with blue color a circle region that has the electron depletion region between the valence and the inner shell. The red color circles indicate the largest portion of the covalent region present between carbon–carbon atoms and carbon–nitrogen atoms. The color shades of the ELF and LOL maps shown in Figure 7 confirm the presence of bonding and non-bonding electrons, where the red color around the hydrogen atoms (H9, H10, H11, H12) with a maximum value show the presence of bonding and non-bonding electrons.

### 3.9. Molecular Reactivity Analysis

**Figure 8:**
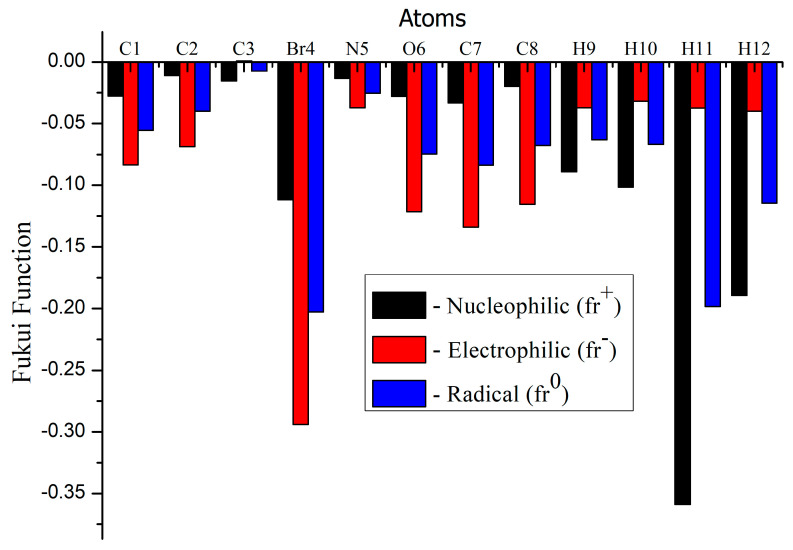
Fukui functions graph of title compound.

**Figure 9:**
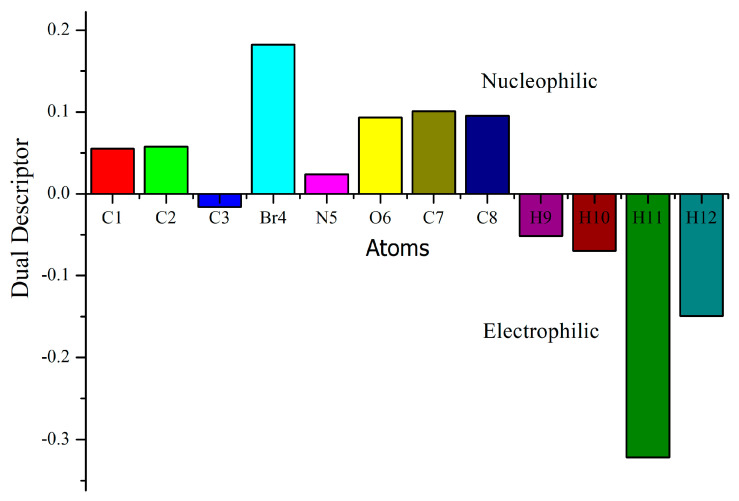
Dual descriptor graph of title compound.

Fukui functions and dual descriptors are a useful guide in analyses of chemical reactivity and site selectivity. The above-mentioned properties are described by using density functional theory, which provides a powerful theoretical framework. In recent years, there has been much attention for molecular reactivity analyses of Fukui functions and dual descriptors using natural population charges (NPA) [71]. The dual descriptor is simply elucidating an electrophilic and nucleophilic character for the title compound as shown in Figure 8 and Figure 9 and Table 6.

The following formulas are used to calculate the Fukui functions and dual descriptor:

(6) $$f^{+}(\vec{r})=q_{r}\left(N+1\right)-q_{r}(N),\;\;\mathrm{for nucleophilic attack}$$

(7) $$f^{-}(\vec{r})=q_{r}\left(N\right)-q_{r}(N-1),\;\;\mathrm{for electrophilic attack}$$

(8) $$f^{0}(\vec{r})=\left(q_{r}\left(N+1\right)-q_{r}(N-1)\right)/2,\;\mathrm{for radical attack}$$

(9) $$∆f\left(\vec{r}\right)=f^{+}\left(\vec{r}\right)-f^{-}\left(\vec{r}\right),\;\;\;\mathrm{(dual descriptor)}$$

The dual descriptor reveals Δƒ($\vec{r}$) > 0 for the nucleophilic attack and Δƒ($\vec{r}$) < 0, the site is preferred for the electrophilic attack. The electrophilic attack of the title compound was observed at C3, H9 and H12 atoms and nucleophilic attacks were identified at C1, C2, Br4, N5, O6, C7 and C8 atoms. The molecular reactivity analyses of Fukui functions and Dual descriptor analyses are in good agreement for MEP surface analyses.

### 3.10. Molecular Docking Analysis

**Figure 10:**
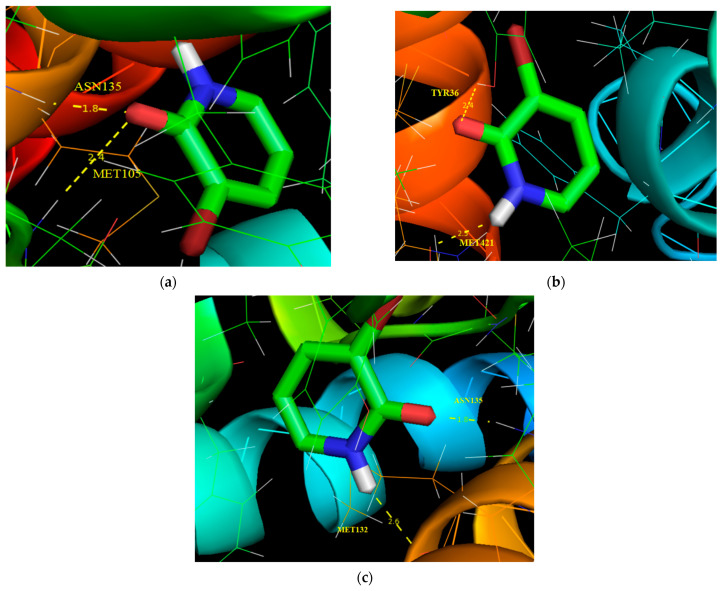
Molecular docking and hydrogen bond interactions of 3-Br-2-HyP with (**a**) 5IBN, (**b**) 3U5K and (**c**) 6CD5 with protein structure.

Molecular docking is an efficacious method to understand bimolecular interactions for rational drug design and discovery. To explore this novel technique, the title compound (ligand) was placed into the preferred binding orientation and affinity of the specific region of the targeted protein (receptor). The BRD (bromodomain inhibitors) have recently been widely focused for the design of pharmacologically active reagents and for cancer treatments [72]. It is probable that BRDs will develop alongside HATs (histone acetyl transferases) and HDAC (histone deacetylases) inhibitors as stimulating targets for drug discovery for the large number of diseases that are produced by unusual acetylation of lysine residues [73]. In the current work, the study compound was chosen with selected proteins in BRD inhibitors using Autodock tools software [74]. The title molecule (ligand) was selected to dock into the active site of the protein chosen from the RCSB PDB [74] format data bank including proteins such as 5IBN, 3U5K and 6CD5. The Autodock tools interface was used to add a polar hydrogen atom, Geisteger charges and Lamarckian genetic algorithm (LGA) calculations were performed in the target proteins. The docking parameters such as binding energy, inhibition constant, bonded residues, bond distance and RMSD values with respect to the selected proteins are listed in Table 7 and their corresponding interaction with selected proteins are shown in Figure 10. The yellow dotted line in Figure 10 represents the bond distance between the intermolecular hydrogen bond lengths and the ligand (title molecule) with respect to protein targets. The minimum binding energy preferred binding orientation in the mode of the docking process. From Table 7, the minimum binding energy of 5IBN, with a binding energy value indicated as −4.71 kcal/mol, had bonded residues as ASN 135, MET 105, respectively.

## 4. Conclusions

In this work, we have carried out a theoretical investigation of 3-bromo-2-hydroxypyridine molecules where there molecular properties have been computed using HF and DFT methods with the 6-311++G(d,p) basis set. The optimized geometry of the studied molecule obtained using the DFT method proves a close accuracy when compared with the Hartree–Fock method. To predict the possibility of the formation of an intermolecular hydrogen bond and to distinguish their nature in the title molecule, AIM calculations have been presented and the analysis of the obtained results are based on the charge density and its Laplacian. For the 3-bromo-2-hydroxypyridine molecule, lower values of the HOMO–LUMO energy gap were observed, which characterize the chemical reactivity and kinetic stability of the molecule. ELF and LOL are used in order to describe the electron distribution and the reactive sites on the surface of the title compound. The UV–Vis spectrum is studied for the gas phase and different solvents: DMSO, water, methanol and chloroform. The evolutions of thermodynamic properties with temperature are given and the correlations were also proposed. The RDG studies are achieved with the aim of elucidating the non-covalent interactions of the present molecule. Reactive sites of the compound were identified from the Fukui functions analysis. The molecular docking shows that the title compound exhibits a BRD inhibitor by the interactions with a suitable protein, with a low binding value of −4.71 kcal/mol. Eventually, if all the results are looked at form head to foot for the 3-bromo-2-hydroxypyridine molecule, they can contribute to the understanding of the structural and physicochemical properties for the pyridine derivatives.

## Figures and Tables

**Table 1:** Monomer and dimer geometrical parameters optimized in 3-Br-2-HyP (bond lengths (Å) and bond angles (°)) by both methods.

Parameters	Monomer				Dimer	
Bond Length (Å)	HF ^a^	DFT ^a^	Theo. ^b^	Exp. ^c^	HF ^a^	DFT ^a^
C1–C2	1.3980	1.4054	1.385	1.406	1.404	1.4131
C1=C3	1.3731	1.3848	1.386	1.378	1.3692	1.3815
C1–Br4	1.8891	1.9053	1.894	1.867	1.8891	1.9043
C2=N5	1.3072	1.3253	1.313	1.334	1.3135	1.3387
C2–O6	1.3259	1.3466	1.338		1.3116	1.322
C3–C7	1.3908	1.3970	1.370	1.375	1.3941	1.3989
C3–H9	1.0742	1.0829	0.930	1.082	1.0742	1.0829
N5–C8	1.3252	1.3387	1.347	1.337	1.3306	1.3429
O6–H10	0.9445	0.9679	0.8		0.9592	1.0073
C7=C8	1.3753	1.3870	1.378	1.38	1.3703	1.3826
C7–H11	1.0731	1.0825	0.93	1.083	1.0728	1.082
C8–H12	1.0756	1.0852		1.087	1.0757	1.085
N5–H22					1.9333	1.7163
H10–N17					1.9329	1.7163
C13–C14					1.404	1.4131
C13=C15					1.3692	1.3815
C13–Br16					1.8891	1.9043
C14=N17					1.3135	1.3387
C14–O18					1.3116	1.322
C15–C19					1.3942	1.3989
C15–H21					1.0742	1.0829
N17–C20					1.3306	1.3429
O18–H22					0.9592	1.0074
C19=C20					1.3703	1.3826
C19–H23					1.0728	1.082
C20–H24					1.0757	1.085
*Bond angle (°)*						
C2–C1=C3	118.0385	118.3261	118.4	118.5	118.5999	119.3112
C2–C1–Br4	121.1088	120.7992			120.6383	120.008
C3=C1–Br4	120.8526	120.8747			120.7618	120.6808
C2–C1–N5	122.6162	122.4936	124.5	123.8	121.5678	120.5763
C1–C2–O6	119.5209	119.6722			119.3431	119.9262
N5=C2–O6	117.8629	117.8341			119.0891	119.4975
C1=C3–C7	119.4275	119.2043	118.4	118.4	119.5604	119.3997
C1=C3–H9	119.7958	119.8381	120.7	120.8	119.7934	119.7699
C7–C3–H9	120.7767	120.9576	120.9	120.8	120.6462	120.8304
C2=N5–C8	119.0116	118.8784	118	116.9	119.4235	119.8116
C2–O6–H10	108.3825	106.6033			111.2939	111.3094
C3–C7=C8	117.6366	118.1407	119.3	118.5	117.4975	117.972
C3–C7–H11	121.2475	120.9465		120.5	121.3459	121.1335
C8=C7–H11	121.1158	120.9128		120.1	121.1566	120.8944
N5–C8=C7	123.2695	122.9568	122.5	123.8	123.3508	122.9291
N5–C8–H12	115.8735	115.906	116.5	116	115.7486	115.7388
C7=C8–H12	120.857	121.1372	121	121.1	120.9005	121.3321
C2=N5–H22					124.2571	123.36
C8–N5–H22					116.3193	116.8283
C14–C13=C15					118.5977	119.312
C14–C13–Br16					120.6394	120.0077
C15=C13–Br16					120.7629	120.6803
C13–C14–N17					121.5697	120.5753
C13–C14–C18					119.3414	119.9295
N17–C14–O18					119.0889	119.4952
C13=C15–C19					119.5615	119.3995
C13=C15–H21					119.7949	119.7704
C19–C15–H21					120.6436	120.8302
H10–N17=C14					124.2639	123.3594
H10–N17–C20					116.3128	116.8286
C14=N17–C20					119.4233	119.8121
C14–O18–H22					111.2924	111.3165
C15–C19=C20					117.4979	117.9715
C14–C19–H23					121.3433	121.1329
C20=C19–H23					121.1588	120.8956
N17–C20=C19					123.3498	122.9296
N17–C20–H24					115.747	115.7384
*Intermolecular H* *−bond lengths (Å) and angles (°)*						
N5·· H22						1.7163
N17·· H10						1.7163
O18–H22						1.0073
O6–H10						1.0073
O6–H10·· N17						174.159
O18–H22·· N5						174.159

^a^ This work; ^b,c^ Values are taken from refs. [13,42].

**Table 2:** AIM parameters of the BCP and RCP for both cyclic dimers of 3-Br-2-HyP.

Parameter	O6–H10	N5··· H22	H10··· N17	RCP_1_	RCP_2_	NRCP
ρ(r)	0.3115	0.0520	0.0520	0.0234	0.0234	0.0081
$\nabla^{2}$ρ(r)	2.1007	0.0998	0.0998	0.1688	0.1688	0.0310
λ_1_	−1.5439	−0.0914	−0.0914	−0.0194	−0.0194	−0.0068
λ_2_	−1.5196	−0.0870	−0.0870	0.0914	0.0914	0.0109
λ_3_	0.9628	0.2783	0.2783	0.0968	0.0968	0.0269
|λ_1_|/λ_3_	1.6035	0.3284	0.3284	0.2004	0.2004	0.2527
[(λ_1_/λ_2_) − 1]	0.5927	0.0107	0.0107	−0.007	−0.0077	−0.0011
H	0.0675	0.0356	0.0356	0.0344	0.0344	0.0066
G	0.6603	0.0463	0.0463	0.0267	0.0267	0.0055
V	0.0160	0.0505	0.0505	−1.2122	−1.2122	−1.6238
G/ρ(r_c_)	0.2169	0.6854	0.6854	1.474	1.474	0.8222

**Table 3:** Calculated energy values for the monomer of 3-Br-2-HyP in gas, water, DMSO and methanol solvents.

Parameters	TD–DFT/B3LYP/6–311++G(d,p)			
	Methanol	Water	DMSO	Gas
E_total_ (Hartree)	−2879.157	−2897.158	−2897.158	−2897.158
E_HOMO_ (eV)	−6.8787	−6.880	−6.880	−6.880
E_LUMO_ (eV)	−1.4751	−1.475	−1.475	−1.475
ΔE_HOMO–LUMO gap_ (eV)	5.404	5.406	5.405	5.406
E_HOMO–1_ (eV)	−7.959	−7.961	−7.960	−7.961
E_LUMO+1_ (eV)	−0.749	−0.749	−0.749	−0.7488

**Table 4:** Calculated parameters of the absorption wavelength (λ), excitation energies (E) and oscillator strengths (*f*) of 3-Br-2-HyP compound using DFT method.

	λ (nm)	E (eV)	*f* (a.u.)	Major Contribution (%)
Gas	260.3	4.76	0.1431	H→L (91)
	240.3	5.15	0.0	H→L + 2 (97)
	234.7	5.28	0.0045	H-2→L (94)
	220.5	5.62	0.0767	H-1→L (33), H→L + 1 (62)
	209.8	5.90	0.0192	H→L+3 (98)
DMSO	260.8	4.75	0.1506	H→L (91)
	240.4	5.15	0.0	H→L+2 (97)
	234.8	5.28	0.0047	H-2→L (94)
	220.8	5.61	0.0807	H-1→L (32), H→L + 1 (63)
	209.8	5.90	0.0196	H→L+3 (98)
Water	260.3	4.76	0.1431	H→L (91)
	240.3	5.15	0.0	H→L+2 (97)
	234.7	5.28	0.0045	H-2→L (94)
	220.5	5.62	0.0767	H-1→L (33), H→L + 1 (62)
	209.8	5.90	0.0192	H→L+3 (98)
Methanol	260.4	4.76	0.1428	H→L (91)
	240.3	5.15	0.0	H→L + 2 (97)
	234.8	5.28	0.0045	H-2→L (94)
	220.6	5.62	0.0765	H-1→L (33), H→L + 1 (62)
	209.8	5.90	0.0191	H→L+3 (98)
Chloroform	261.9	4.73	0.1541	H→L (91)
	240.4	5.15	0.0	H→L+2 (97)
	236.0	5.25	0.0047	H-1→L (93)
	221.7	5.59	0.0823	H-2→L (32), H→L + 1 (63)
	210.4	5.89	0.0193	H→L + 3 (97)

**Table 5:** Calculated thermodynamic properties for 3-Br-2-HyP compound.

T (K)	S (J/mol·K)	C_p_ (J/mol·K)	H (kJ/mol)
100	267.79	50.60	3.90
200	311.88	80.65	10.44
298.15	349.82	111.23	19.86
300	350.51	111.79	20.07
400	386.63	139.91	32.69
500	420.43	163.00	47.88
600	451.82	181.26	65.13
700	480.89	195.68	84.00
800	507.80	207.25	104.17
900	532.78	216.71	125.38
1000	556.03	224.57	147.46

**Table 6:** Natural population distribution, Fukui functions and dual descriptor of the title compound.

Atom	Natural Charges			Fukui Functions			Dual Descriptor
	0, 1 (N)	N + 1 (−1, 2)	N − 1 (1, 2)	fr^+^	fr^−^	fr^0^	Δf
C1	−0.18612	−0.21400	−0.10282	−0.02788	−0.08330	−0.05559	0.05542
C2	0.50801	0.49680	0.57671	−0.01121	−0.06870	−0.03996	0.05749
C3	−0.17061	−0.18618	−0.17137	−0.01557	0.00076	−0.00741	−0.01633
Br4	0.09704	−0.01467	0.39100	−0.11171	−0.29396	−0.20284	0.18225
N5	−0.51337	−0.52676	−0.47605	−0.01339	−0.03732	−0.02536	0.02393
O6	−0.65520	−0.68317	−0.53381	−0.02797	−0.12139	−0.07468	0.09342
C7	−0.26329	−0.29652	−0.12926	−0.03323	−0.13403	−0.08363	0.10080
C8	0.06479	0.04476	0.18025	−0.02003	−0.11546	−0.06775	0.09543
H9	0.22433	0.13549	0.26162	−0.08884	−0.03729	−0.06307	−0.05155
H10	0.48401	0.38233	0.51591	−0.10168	−0.03190	−0.06679	−0.06978
H11	0.21809	−0.14101	0.25560	−0.35910	−0.03751	−0.19831	−0.32159
H12	0.19233	0.00292	0.23223	−0.18941	−0.03990	−0.11466	−0.14951
C1	−0.18612	−0.21400	−0.10282	−0.02788	−0.08330	−0.05559	0.05542
C2	0.50801	0.49680	0.57671	−0.01121	−0.06870	−0.03996	0.05749

**Table 7:** Hydrogen bonding and molecular docking with protein targets.

Protein (PDB ID)	Bonded Residues	Bond Distance (Å)	Estimated Inhibition Constant (μm)	Binding Energy (kcal/mol)	Reference RMSD (Å)
5IBN	ASN135	1.8	351.98	−4.71	17.207
	MET105	2.4			
3U5K	TYR36	2.4	454.17	−4.56	79.38
	MET421	2.5			
6CD5	ASN135	1.8	757.63	−4.26	14.506
	MET132	2.6			

## Data Availability

Not applicable.

## Supplementary Materials

The following supporting information can be downloaded at: https://www.mdpi.com/article/10.3390/molecules28062669/s1, Figure S1: Correlation graphic between the calculated and (a) theoretical bond lengths, (b) experimental bond lengths, (c) theoretical bond angles and (d) experimental bond angles; Figure S2: Variation of entropy (S), heat capacity (Cp) and enthalpy (H) with temperature for the 3-Br-2-HPy molecule; Table S1: Calculated SCF energy, ZPVE, rotational constants, thermodynamic parameters and dipole moment (D) of 3-Br-2-HyP monomer and dimer structures with DFT method.
