# Supplementary material for: Molecular Structure, Spectral Analysis, Molecular Docking and Physicochemical Studies of 3-Bromo-2-hydroxypyridine Monomer and Dimer as Bromodomain Inhibitors
Source: Molecules. 2023 Mar 15;28(6):2669. doi: 10.3390/molecules28062669 (PMC10054851; doi:10.3390/molecules28062669)
Supplement: Supplementary file 1 [file molecules-28-02669-s001.zip › molecules-2189273-supplementary.pdf]

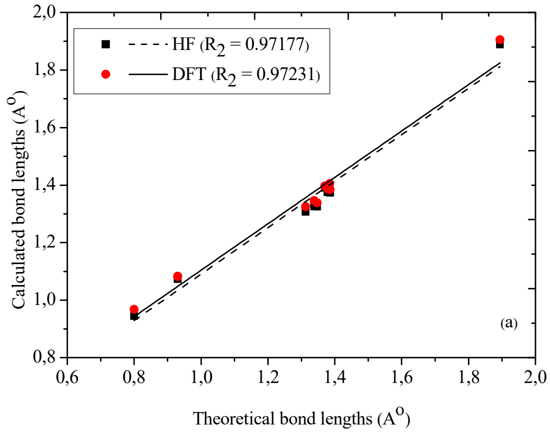

(**a**)

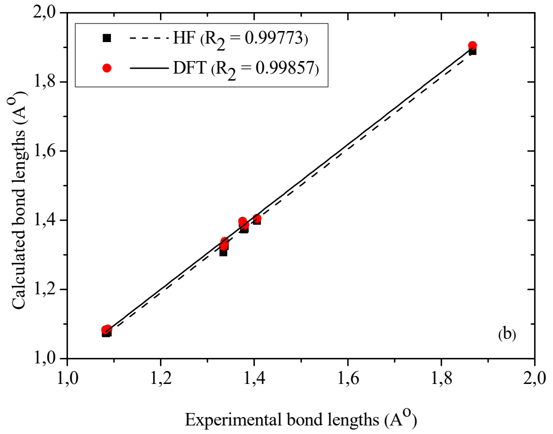

(**b**)

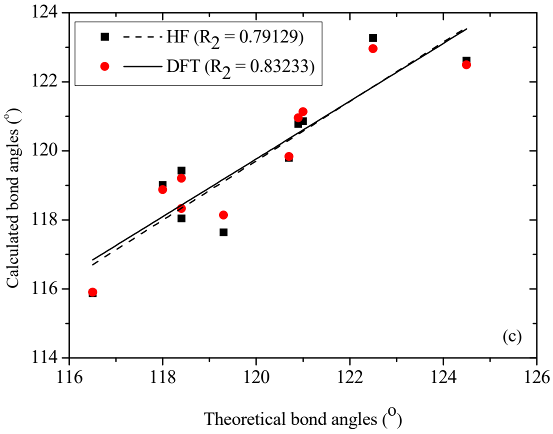

(c)

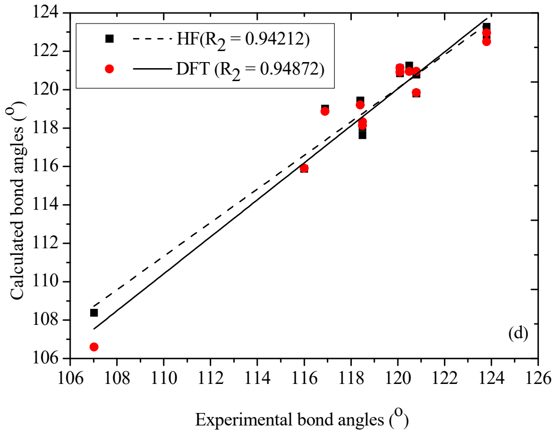

(d)

Figure S1. Correlation graphic between the calculated and ((a) and (b) theoretical and experimental bond lengths (c)), and ((d) theoretical and experimental bond angles).

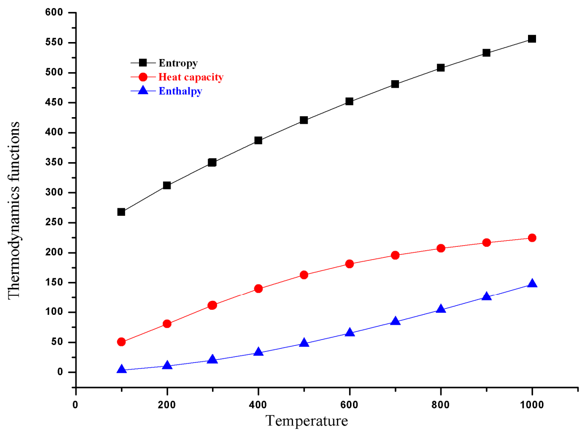

Figure S2. Variation of entropy (S), heat capacity (Cp) and enthalpy (H) with temperature for the 3-Br-2-HPy molecule.

Table S1. Calculated SCF energy, ZPVE, rotational constants, thermodynamic parameters and dipole moment (D) of 3-Br-2-HyP monomer and dimer structures with DFT method.

	**Monomer**	**Dimer**
**SCF energy E(kJ/mol)**	−7606473.060	−15213012.632
**ZPVE (kcal/mol)**	52.10659	104.91781
**Rotational constants (GHz)**		
**A**	2.97625	0.71148
**B**	0.98182	0.11026
**C**	0.73827	0.09547
**Thermal energy (kcal/mol)**	56.262	114.086
**Specific heat at constant Volume C_v (cal.mol^{-1}.K^{-1})**	24.598	52.683
**Entropy S (cal.mol^{-1}.K^{-1})**	83.583	128.527
**Dipole moment (Debye)**	2.1152	0.0019
